# Effects of Undernutrition and Hydroxytyrosol Supplementation in Late Pregnancy on Growth and Metabolic and Endocrine Profiles of Male Beef Offspring During the Fattening Phase

**DOI:** 10.3390/ani16131993

**Published:** 2026-06-28

**Authors:** Olaia Akesolo-Atutxa, Leire López de Armentia, Agustí Noya, Guillermo Ripoll, Javier Álvarez-Rodríguez, Albina Sanz

**Affiliations:** 1 Departamento de Ciencia Animal, Centro de Investigación y Tecnología Agroalimentaria de Aragón (CITA), Avda. Montañana 930, 50059 Zaragoza, Spain; llopezdearmentia@cita-aragon.es (L.L.d.A.); gripoll@cita-aragon.es (G.R.); 2 Instituto Agroalimentario de Aragón—IA2 (CITA-Universidad de Zaragoza), C/Miguel Servet 177, 50013 Zaragoza, Spain; javier.alvarezr@unizar.es; 3 Departament Ciència Animal, Universitat de Lleida, Avda. Rovira Roure 191, 25198 Lleida, Spain; agusti.noya@udl.cat; 4 Departamento de Producción Animal y Ciencia de los Alimentos, Escuela Politécnica Superior de Huesca, Carretera de Cuarte s/n, 22071 Huesca, Spain

**Keywords:** fattening phase, fetal programming, performance, antioxidants, metabolism, prenatal nutrition

## Abstract

Maternal undernutrition in late gestation can compromise fetal development and postnatal productivity in beef cattle. This study evaluated whether maternal supplementation with hydroxytyrosol (HT)—a potent antioxidant from olive by-products—during late gestation could alleviate the effects of undernutrition on growth, concentrate conversion ratio, and metabolic and endocrine adaptation of male offspring during the fattening phase. Forty-six multiparous pregnant cows were assigned to four dietary treatments combining nutritional level (T100% vs. T60% of nutritional requirements) and HT supplementation (CONTROL vs. HT at 180 mg/kg of diet) exclusively during the last third of pregnancy. Maternal undernutrition reduced calf morphometric development at the beginning of fattening (4 months), although compensatory growth enabled recovery by slaughter (12 months). For live weight (LW), the feeding level and HT interaction showed a tendency (*p* = 0.084), but pairwise comparisons within the restricted groups suggested that calves from HT-supplemented dams were 31 kg heavier at slaughter than calves from non-supplemented dams (*p* = 0.023). HT supplementation was also associated with higher plasma urea concentrations at 12 months in restricted calves, suggesting potential adaptations in nitrogen metabolism without changes in glucose or IGF-1 profiles. In summary, these findings might support our hypothesis suggesting potential benefits in the development of male offspring from HT-supplemented cows during the last third of gestation. However, further studies will be needed to demonstrate definitive overall evidence of HT benefits.

## Simple Summary

Maternal undernutrition during the final stage of pregnancy can impair calf development and reduce productivity in beef cattle systems. This study evaluated whether supplementing undernourished pregnant cows with hydroxytyrosol, a natural antioxidant derived from olive by-products, could mitigate these adverse effects. Calves born to undernourished mothers were smaller at the beginning of the fattening phase but, afterwards, they were able to recover their growth when provided with adequate feeding. Although the antioxidant did not show an overall effect, a difference of 31 kg in final live weight was observed between calves from undernourished cows that received hydroxytyrosol compared to those calves from unsupplemented undernourished cows. Punctual weight differences might support our hypothesis suggesting a possible benefit of hydroxytyrosol supplementation in offspring development under challenging prenatal conditions. This approach explores a practical and sustainable strategy utilizing agricultural by-products to potentially enhance beef production. However, further studies will be needed to demonstrate definitive overall evidence of HT benefits.

## 1. Introduction

In beef cattle production systems, inadequate maternal nutrition remains a critical challenge, particularly in extensive systems where feed availability depends on fluctuating pasture availability and limited forage resources. Although fetal growth is continuous throughout gestation, it is well established that over 75% of total fetal weight gain occurs in the last two months of gestation when the fetus nutritional demands become quantitatively significant relative to the dam’s requirements [1]. In bovine fetuses, the total number of muscle fibers is fixed by day 240 of gestation, and this quantity, together with fiber type and size, is a dominant factor in postnatal muscle mass. Moreover, total fiber number is positively correlated with growth potential [2,3], so an alteration of fetal tissue differentiation as a consequence of inadequate maternal nutrition during late gestation may have detrimental effects on fetal growth and muscle development [4,5], potentially affecting calf development after birth [6].

One key mechanism underlying the developmental consequences of inadequate maternal nutrition is intrauterine growth restriction (IUGR), which is often associated with a weakened antioxidant defense system in the fetus [7,8,9]. Several studies have shown that antioxidant supplementation can improve the antioxidant/oxidative balance, thereby protecting placental function and the fetoplacental unit from oxidative damage [10,11,12,13]. Polyphenols constitute an abundant group of natural antioxidants and chemopreventive agents widely distributed in plants [14]. Their ingestion has been shown to enhance plasma antioxidant capacity and reduce placental oxidative stress [15,16,17], thereby improving pregnancy outcomes and reducing the incidence of IUGR [18,19,20]. Among polyphenols, hydroxytyrosol (HT), a compound found in olive by-products [21], has been recognized for its potent antioxidant, anti-inflammatory, metabolic-regulatory, and immunomodulatory properties [21,22,23]. In a pig model, maternal supplementation with HT improved fetal oxidative status and reduced the risk of low birth weight [24,25]. Moreover, progeny from HT-supplemented sows exhibited enhanced growth, body size, muscle development, and adiposity during the fattening phase compared to controls [24].

The impact of maternal undernutrition during gestation on subsequent offspring development remains controversial: while several studies report detrimental effects on growth, metabolism, and body composition [26,27,28,29,30], others observe compensatory growth or attenuated differences, especially when postnatal nutrition is adequate [27,28,29]. These discrepancies may reflect differences in breed, timing, severity, and duration of undernutrition, as well as in the postnatal environment. Evidence regarding the effects of HT supplementation in beef cattle is scarce. While previous research from our group on the same herd evaluated the effects of maternal HT supplementation in the last third of gestation on the dams and the early postnatal period of the calves [31,32,33], its potential long-term consequences during the fattening phase have not yet been investigated. Addressing this knowledge gap, the present study provides novel evidence by evaluating whether maternal HT supplementation during late gestation can mitigate the effects of maternal undernutrition on the growth performance and metabolic adaptation of male offspring throughout the fattening phase (4–12 months of age). In the Spanish beef production system, bull calves slaughtered between 8 and 12 months of age represent a highly demanded carcass category, accounting for more than 30% of the total bovine carcasses, which clearly differentiates the Spanish beef sector from other EU countries where most carcasses correspond to yearlings (12–24 months) [34].

Therefore, this study aimed to analyze the effects of maternal undernutrition and dietary HT supplementation during the last third of gestation on concentrate intake, concentrate conversion ratio, growth, body size, and metabolic (glucose, fructosamine, urea, creatinine, non-esterified fatty acid (NEFA)) and endocrine (insulin-like growth factor 1 (IGF-1)) profiles of male offspring during the fattening phase (4–12 months of age). This work expands current knowledge on the long-term consequences of prenatal nutritional management in beef cattle and provides novel evidence that maternal HT supplementation may partially modulate the developmental programming effects associated with gestational undernutrition, particularly regarding postnatal growth performance.

## 2. Materials and Methods

### 2.1. Animal Ethics

The experiment was conducted in accordance with the stipulations set out in the Spanish Policy for animal protection RD 53/2013, which aligns with the European Union Directive 2010/63/EU on the protection of animals used for experimental and other scientific purposes. Furthermore, the animal procedure protocol was approved by the in-house Ethical Committee of the Centro de Investigación y Tecnología Agroalimentaria de Aragón (CITA), as evidenced by protocol number CEEA-04 2021-09.

### 2.2. Animal Management

#### 2.2.1. Cow Management

Cow gestation and lactation phases of the suckling calves were conducted at CITA-La Garcipollera Research Station, in the mountain area of the central Pyrenees (Huesca, Spain, 945 m a.s.l.). For the present study, a subset of 46 multiparous Parda de Montaña (PA; *n* = 25) and Pirenaica (PI; *n* = 21) cows was selected from a larger experimental suckler cattle herd previously described in López de Armentia et al. [32]. Cows were randomly inseminated with semen from one of two registered sires per breed, which were equally represented across the subsequent maternal nutritional treatments, and those cows diagnosed as carrying male fetuses were included in the present experiment.

During the first and second thirds of pregnancy (weeks 0 to 28 of gestation), all pregnant dams received a complete diet to meet their maintenance, lactation, and gestation requirements. From week 28 of gestation to calving (week 40), dams received a forage-based total mixed ration (TMR) and were allocated to four dietary treatments following a 2 × 2 factorial design. The factors were feeding level according to INRA [35] (100% vs. 60% of maintenance and gestational nutrient requirements) and HT supplementation in the diet (CONTROL vs. HT, corresponding to 0 and 180 mg HT/kg TMR, respectively). Prior to treatment allocation, cows were weighed and their body condition score (BCS) was recorded. All groups were balanced in LW, BCS, breed and age. This resulted in the following groups: T100%-CONTROL (*n* = 10), T100%-HT (*n* = 11), T60%-CONTROL (*n* = 13), and T60%-HT (*n* = 12). At the beginning of the experiment, cows had an average LW of 657 ± 6.98 kg, a BCS of 3.5 ± 0.06 (on a scale of 1 to 5), and were 8.71 ± 0.35 years old.

Specifically, this TMR was composed of barley straw (49.6%), barley grain (24.8%), alfalfa pellet (8.4%), rapeseed meal (6.9%), sugar-beef pulp (4.5%), soybean meal (2.5%), calcium carbonate (0.80%), di-calcium phosphate (0.25%), sodium chloride (0.25%), vitamin-micro mineral corrector (0.20%), and water (for CONTROL groups) or HT solution (for HT groups) (1.8%). Hydroxytyrosol was added as a liquid solution (18 L/t of diet) using a commercial preparation containing 100 g/L with 10% pure HT (Econatur, Córdoba, Spain), resulting in a final supplementation level of 180 mg HT/kg of diet (on an as-fed basis). This dosage was determined based on the manufacturer’s recommendations and previous evidence of its health-promoting effects in monogastric species [36,37,38]. Given the absence of prior data regarding HT supplementation in ruminants, this concentration was selected to guarantee an effective systemic delivery while remaining safely below the No Observed Adverse Effect Level (NOAEL) for subchronic toxicity established by the European Food Safety Authority (EFSA) [39]. The CONTROL diet included the same amount of solution in water form (18 L/t of diet). The solutions were thoroughly mixed into the TMR using a mixer wagon to ensure a homogeneous distribution. Although the chemical stability of HT directly within the feed bunk was not assessed, the proper ingestion, absorption, and bioavailability of the compound were confirmed in vivo by the detection of specific HT phase II metabolites (hydroxytyrosol sulphate and alcohol homovanillic sulphate) in the blood serum and colostrum of the supplemented dams, as detailed in previous reports from this experimental herd [31,32]. The TMR did not differ between dietary treatments, except in HT content. The final dry matter (DM) content of the TMR was 888 g/kg of feed. The macronutrient supply of the TMR was 111 g of CP/kg DM, 161 g of starch/kg DM, 529 g of neutral-detergent fiber/kg DM, 328 g of acid-detergent fiber/kg DM, 53 g of acid-detergent lignin/kg DM, and 19 g of ether extract/kg DM. According to the INRA nutritive value calculations [35], the estimated energy value was 1240 kcal/kg DM of net energy for lactation.

The T100% cows received 10.5 kg of TMR/day, while the T60% cows received 7 kg of the same TMR/day. During the subsequent lactation period, all dams were fed 100% of their requirements (10.5 kg of TMR/day) using the TMR described above, and without HT supplementation. Throughout the study, the cows had access to mineral lick blocks, which they consumed at approximately 75–80 g/day.

Regarding housing, cows were distributed into four loose-housed pens (one pen per dietary treatment group) where they were group-fed during the last third of gestation. To minimize competition and ensure equitable individual intake, cows were restrained at the feeder using self-locking headlocks for 3 h immediately after feed distribution. After parturition, they remained with their calves for the first 12 h, then were separated into loose-housed pens that allowed cow–calf fence contact. Calves were fed exclusively colostrum and maternal milk in a restricted, twice-daily nursing system and were weaned at 4 months of age.

#### 2.2.2. Management of the Male Offspring During the Fattening Phase

Once weaned, a total of 46 bull calves (*n* = 10 for T100%-CONTROL, *n* = 11 for T100%-HT, *n* = 13 for T60%-CONTROL, and *n* = 12 for T60%-HT) were transported for fattening to the CITA-Montañana Research Station (Zaragoza; 41°43′ N, 0°48′ W; 230 m a.s.l.), which is located in the Ebro Valley (Spain), an area with a continental Mediterranean climate. The experiment was carried out from June 2022 to January 2023. Bull calves were housed under natural conditions (maximum, mean and minimum of ambient temperatures of 30.0, 16.3 and 4.5 °C; maximum, mean and minimum of ambient light 462, 367 and 286 daylight hours/month). Ventilation was provided through natural airflow assisted by lateral openings to ensure adequate air renewal. Bull calves were distributed in different pens according to their prenatal feeding level (T100% vs. T60%). Due to space limitations at the facilities, it was not possible to allocate calves into pens according to the full 2 × 2 factorial design. Consequently, calves from both CONTROL and HT groups were randomly mixed within each maternal feeding level. Each prenatal feeding level was replicated four times, resulting in a total of eight calf group pens with 5–6 animals per pen (space allowance ranged from 4.6 to 5.5 m^2^ per calf, and straw as bedding material). The transition period lasted 7 days, during which they received a diet with increasing amounts of barley straw and a commercial concentrate for growing cattle, provided as a mash (Table 1). After this transition period (day 0), throughout the entire fattening phase, all bull calves were fed ad libitum with the same commercial concentrate plus barley straw. In the present study, straw intake was not recorded; in this sense, in Spanish concentrate-based bull-fattening systems, cereal straw typically represents approximately 5–10% of total feed intake [40]. The chemical composition of concentrate and barley straw, and their net energy values estimated according to the FEDNA tables [41], are presented in Table 2. Water was supplied ad libitum in individual bowl-cup drinkers. This management aimed to produce carcasses of entire males of 12 months of age, which constitute one of the most prevalent carcass categories in Spain.

### 2.3. Performance of the Male Offspring During the Fattening Phase

#### 2.3.1. Growth, Feed Intake, and Development of Bull Calves

Bull calves were weighed every month, and the average daily gain (ADG) for each animal was estimated by linear regression using all monthly LW measurements. The variables for concentrate intake and concentrate conversion ratio were only evaluated considering maternal feeding level (T100% vs. T60%); concentrate intake (offer minus refusals) was recorded daily per group, and mean pen values were used to calculate the pen concentrate conversion ratio. Dietary nitrogen (N) excretion was calculated as consumed N (in function of concentrate intake and its N content) minus retained N (estimated from body weight gain, assuming that nitrogen constitutes 16% of the deposited crude protein, based on the standard conversion equation: Crude Protein = N × 6.25), according to MAPA [42]. Nitrogen use efficiency was defined as retained N/consumed N. Size measurements were taken at the start and end of the fattening phase (at 4 and 12 months of age, respectively) to assess body growth. Height at withers (distance from the floor to the highest point of the withers), height at rump (distance from the floor to the highest point of the internal tuberosity of ilium), rump width (maximum distance between iliac tuberosities), rump length (distance from the ischial tuberosity to the external iliac tuberosity), body length (distance from the cranial side of the shoulder blades to the caudal side of the ischial tuberosity), chest girth (circumference immediately behind the shoulder blades in a plane perpendicular to the body axis), head girth (measurement collected around parietal bone and mandible just posterior to eye orbits) and metacarpal girth (narrowest point of metacarpus) were the variables evaluated. The bull calf was standing naturally, head up, and with weight on all four feet when all body measures were obtained. Measurements of the bull calf’s head were made while it was under restraint. The body mass index (BMI) of bull calves was calculated by dividing the live weight (kg) of each bull calf by the square root of body length (cm), following the methodology described by Maresca et al. [43] for detecting disproportionate fetal growth.

#### 2.3.2. Metabolic and Endocrine Profiles

Blood samples were collected every two months (at 4, 6, 8, 10, and 12 months of age) at 08:00 by coccygeal venipuncture into heparinized and EDTA tubes (BD Vacutainer Becton-Dickenson and Company, Plymouth, UK) to measure the concentrations of glucose, fructosamine, NEFA, urea, creatinine, and IGF-1 to evaluate the metabolic and endocrine status of bull calves. Bull calves had ad libitum access to concentrate, so a fasting state could not be imposed. LW and blood samplings were strictly performed at the same time of day to minimize diurnal and handling-induced variations. Heparinized plasma was used to measure creatinine, urea, and IGF-1 concentrations, whereas EDTA plasma was used to measure glucose, fructosamine, and NEFA. Following collection, samples were centrifuged at 1500× *g* for 20 min at 4 °C. The plasma was then extracted and stored at −20 °C until analysis. Additionally, the urea/creatinine (U/C) ratio was calculated for each sampling point by dividing the plasma urea concentration by the plasma creatinine concentration.

### 2.4. Assays

An automatic analyzer (BA 400 Led technology BioSystems, Barcelona, Spain) was used to measure blood concentrations of glucose (glucose oxidase/peroxidase method, sensitivity: 0.199 mmol/L), urea (kinetic UV test, sensitivity: 0.167 mmol/L), creatinine (enzymatic method, sensitivity: 0.088 mmol/L) and fructosamine (nitroblue tetrazolium (NBT) method, sensitivity: 0.14 mmol/L). Insulin-like growth factor 1 (enzyme immunoassay; sensitivity: 14.4 ng/mL) was measured using a solid-phase, enzyme-labeled chemiluminescent immunometric assay (Immulite; Siemens Medical Solutions Diagnostics Limited, Llanberis, Gwynedd, UK). Intra-assay coefficients of variation for all commercial serum controls were ≤10%. Non-esterified fatty acids (enzymatic method, sensitivity: 0.07 mmol/L) were analyzed using a commercial kit (Randox Laboratories Ltd., Crumlin Co., Antrim, UK). The mean intra-assay coefficients of variation were 4.74% and 4.81% for high and low controls, respectively, and the mean inter-assay coefficients of variation were 4.51% and 4.32% for high and low controls, respectively.

### 2.5. Statistical Analysis

All statistical analyses were performed using R software (version 4.4.2; R Core Team, Vienna, Austria) [44]. The normality of the model residuals was assessed using the Shapiro–Wilk test (*p* > 0.05). Overall ADG for the entire fattening phase, bull calf body size measures, duration of the fattening phase, and age at slaughter were analyzed with generalized linear models using the glm function with maternal feeding level (T100% vs. T60%), HT supplementation (CONTROL vs. HT), their interaction, and breed (PA vs. PI) as fixed effects. Bull calf LW, monthly ADG, concentrate intake, concentrate conversion ratio, nitrogen excretion, nitrogen use efficiency, and metabolite and hormone concentrations (glucose, fructosamine, NEFA, urea, creatinine, urea/creatinine ratio, IGF-1) were analyzed using linear mixed-effects models using the lmer function with the lmerTest R package (v3.1.3; [45]) for repeated measures, applying Satterthwaite’s degrees of freedom method. Maternal feeding level, HT supplementation and breed (between-subject effects); the sampling month (within-subject effect); and their interactions were analyzed as fixed effects. A compound symmetry covariance structure was assumed for the repeated-measures models by fitting the respective experimental unit as the random effect. Specifically, the individual animal was considered the experimental unit for all individual traits (overall ADG, body size, LW, monthly ADG, nitrogen efficiency traits, and metabolic/endocrine traits). For the repeated-measures variables analyzed with mixed models (LW, monthly ADG, nitrogen efficiency traits, and metabolic/endocrine traits), initial exploratory models evaluated the inclusion of the pen as an additional random effect. However, because all pens were located adjacent to each other in a single enclosed barn under highly uniform environmental conditions, pen-associated variability was considered negligible. Therefore, to avoid model overparameterization, the pen effect was excluded from the final mixed models, retaining the individual animal as the sole experimental unit. Conversely, since concentrate intake could only be recorded per group, the pen was considered the experimental unit for concentrate intake and concentrate conversion ratio (*n* = 4 pens per maternal feeding level), meaning the main effect of HT and its interaction on these variables could not be assessed. Least squares means were estimated for each fixed effect, and pairwise comparisons of the means were obtained using the emmeans R package (v1.10.5; [46]), applying Fisher’s Least Significant Difference test without further adjustments for multiple comparisons. Initial LW of the dam at week 28 of gestation, nested within breed, was included as a covariate in all analyses. The relationships among the variables were determined using Pearson’s correlation coefficients. A significant difference was defined as *p* < 0.05, and a tendency was defined as *p* < 0.10. The results are presented as least square means ± standard error (SE) in the text and as least square means with the residual standard deviation (RSD) in the tables.

## 3. Results

### 3.1. Concentrate Intake and Concentrate Conversion Ratio

**Figure 1:**
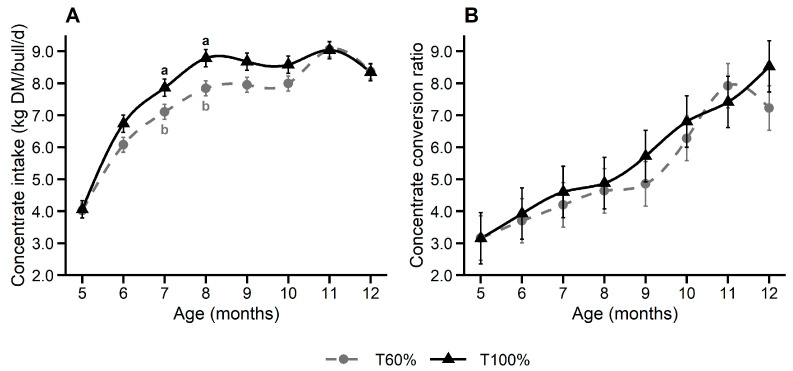
(**A**) Concentrate intake and (**B**) concentrate conversion ratio during the fattening phase (4–12 months of age) in bull calves, according to maternal feeding level (FL) during the last third of gestation. Different letters (a, b) between treatments indicate significant differences (*p* < 0.05). The experimental unit was the pen (*n* = 4 pens per maternal FL). DM = dry matter; T60% = bull calves from cows fed 60% of their nutritional requirements; T100% = bull calves from cows fed 100% of their nutritional requirements.

The evolution of concentrate intake and concentrate conversion ratio during the fattening phase, as influenced by the interaction between maternal feeding level and age, is shown in Figure 1. Concentrate intake increased sharply between 5 and 8 months of age and then remained relatively stable until slaughter, being higher in T100% than in T60% bull calves at 7 and 8 months of age (*p* < 0.05). Concentrate conversion ratio increased progressively with age in both groups and did not differ significantly between maternal feeding levels at any specific month (*p* > 0.05). However, the average concentrate intake across the whole fattening phase tended to be lower in T60% than in T100% bull calves (6.14 ± 0.12 vs. 6.53 ± 0.15 kg DM/bull/day, *p* = 0.09) and, in the same line, the average concentrate conversion ratio also tended to be lower in T60% than in T100% bull calves (4.50 ± 0.09 vs. 4.84 ± 0.10, *p* = 0.06). Overall, the T60% bull calves showed greater nitrogen use efficiency (retained N/consumed N) than their T100% counterparts during the whole fattening phase (0.269 vs. 0.248 ± 0.006, *p* < 0.05), regardless of maternal HT supplementation (*p* > 0.10). The aforementioned difference was more marked from 6 to 10 months of age, leading to lower N excretion in the T60% bull calves during this time frame (*p* < 0.05).

### 3.2. Bull Calf Growth and Development

**Figure 2:**
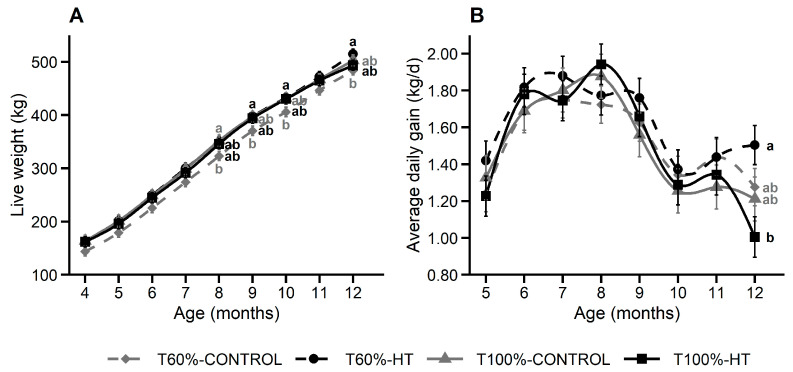
(**A**) Live weight and (**B**) average daily gain during the fattening phase (4–12 months of age) in bull calves, according to maternal feeding level (FL) and hydroxytyrosol supplementation (HT) during the last third of gestation. Different letters (a, b) indicate significant differences among the four treatment groups within the same age (*p* < 0.05). Sample sizes were *n* = 13 for T60%-CONTROL, *n* = 12 for T60%-HT, *n* = 10 for T100%-CONTROL, and *n* = 11 for T100%-HT. T60%-CONTROL = bull calves from cows fed 60% of their requirements and not supplemented with HT; T60%-HT = bull calves from cows fed 60% of their requirements and supplemented with HT; T100%-CONTROL = bull calves from cows fed 100% of their requirements and not supplemented with HT; T100%-HT = bull calves from cows fed 100% of their requirements and supplemented with HT.

Bull calf LW and ADG during the fattening phase are presented in Figure 2. Age had a strong effect on both LW and ADG (*p* < 0.001), whereas the three-way interaction between maternal feeding level, HT supplementation, and age was not significant for either trait (*p* > 0.05). During the first half of the fattening phase (4–8 months of age), LW and ADG were similar between groups. From 8 months of age onwards, T60%-HT bull calves tended to be heavier than T60%-CONTROL bull calves, with significant differences in LW observed at 9, 10, and 12 months of age (*p* < 0.05). At the end of the fattening phase (12 months of age), T60%-HT bull calves weighed 31 kg more than T60%-CONTROL bull calves (*p* = 0.023), whereas no differences were observed between T100%-HT and T100%-CONTROL bull calves or between T100%-HT and T60%-HT bull calves (*p* > 0.05). Monthly ADG was generally similar between groups throughout the fattening phase; however, in the last month (11–12 months of age), T60%-HT bull calves showed a higher ADG than T100%-HT bull calves (*p* < 0.01). In contrast, no other pairwise differences were detected at this time. Overall growth performance variables are summarized in Table 3. No significant effects of maternal feeding level, HT supplementation, or their interaction were observed for LW at the start of the fattening phase, or ADG over the whole fattening phase (*p* > 0.05). Regarding final LW at slaughter, the global model revealed a tendency for the interaction between maternal feeding level and HT supplementation (*p* = 0.084). However, based on the a priori hypothesis that HT supplementation could mitigate the impact of prenatal undernutrition, specific pairwise comparisons were evaluated, revealing the aforementioned 31 kg difference between T60%-HT and T60%-CONTROL bull calves (*p* = 0.023). Similarly, no differences were detected for the duration of the fattening phase or age at slaughter (*p* > 0.05).

Body size measures during the fattening phase are presented in Table 4. Height at rump (*p* = 0.015), body length (*p* = 0.027), chest girth (*p* = 0.004), and head girth (*p* = 0.019) at the start of fattening were affected by maternal feeding level, with T60% bull calves showing lower values than T100% bull calves (*p* < 0.05). However, at the end of the fattening phase (12 months of age), no differences in these body measurements were observed between groups (*p* > 0.05). Metacarpal girth was affected by the interaction between maternal feeding level and HT at the start of fattening (*p* = 0.007); it was larger in the T100%-CONTROL and T60%-HT groups than in the T100%-HT group (*p* < 0.05), with no differences at the end of fattening (*p* > 0.05). Regarding the breed, PA bull calves presented higher values than PI in metacarpal girth (both at the start (*p* = 0.007) and at the end (*p* = 0.003) of fattening), rump width (at the end of fattening, *p* = 0.020) and chest girth (at the end of fattening, *p* = 0.005). Bull calf BMI at the start of fattening was lower in T60% than in T100% bull calves and in PI than in PA bull calves (16.3 ± 0.36 vs. 15.3 ± 0.32 kg/cm^0.5^ for T100% and T60%, and 16.4 ± 0.32 vs. 15.2 ± 0.36 kg/cm^0.5^ for PA and PI, respectively; *p* < 0.05), while no significant differences were observed at the end of fattening.

### 3.3. Metabolic and Endocrine Profiles

**Figure 3:**
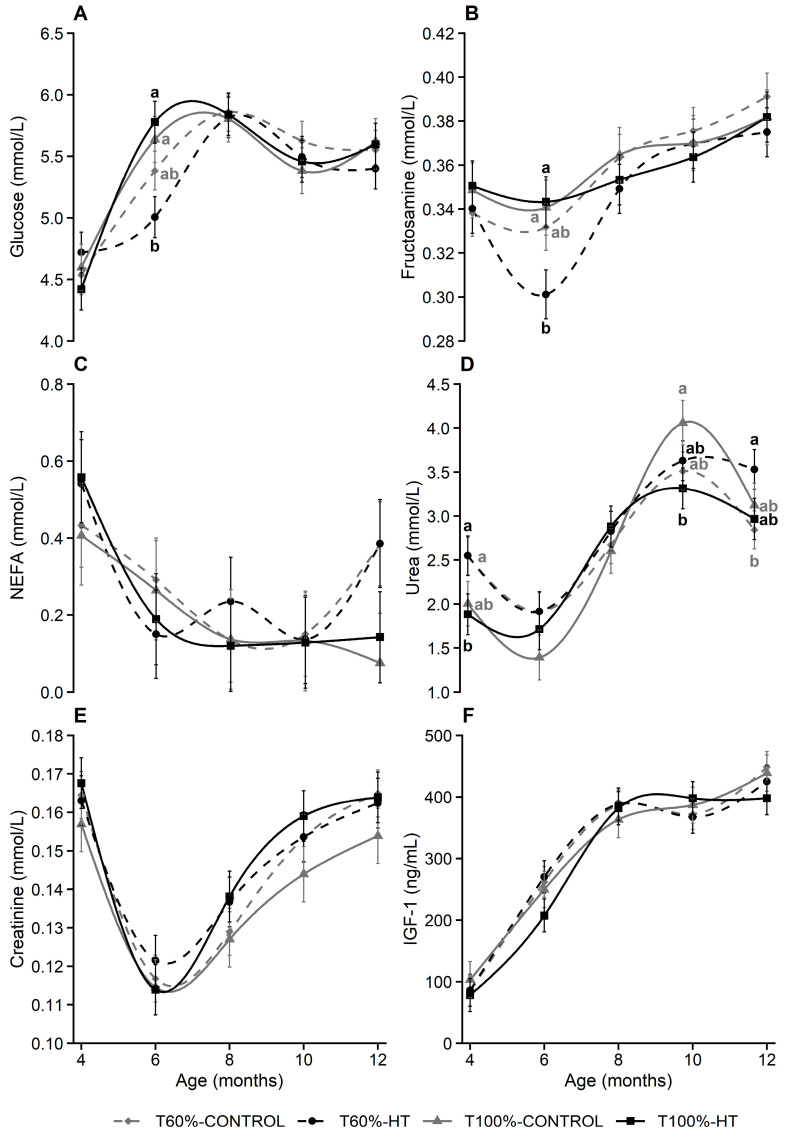
Plasma metabolite and hormone concentrations during the fattening phase (4–12 months of age) in bull calves, according to maternal FL and HT supplementation during the last third of gestation: (**A**) glucose, (**B**) fructosamine, (**C**) NEFA, (**D**) urea, (**E**) creatinine, and (**F**) IGF-1. Different letters (a, b) indicate significant differences among the four treatment groups within the same age (*p* < 0.05). Sample sizes were *n* = 13 for T60%-CONTROL, *n* = 12 for T60%-HT, *n* = 10 for T100%-CONTROL, and *n* = 11 for T100%-HT. FL = feeding level; HT = hydroxytyrosol; T60%-CONTROL = bull calves from cows fed 60% of their requirements and not supplemented with HT; T60%-HT = bull calves from cows fed 60% of their requirements and supplemented with HT; T100%-CONTROL = bull calves from cows fed 100% of their requirements and not supplemented with HT; T100%-HT = bull calves from cows fed 100% of their requirements and supplemented with HT.

The concentrations of metabolites and hormones during the fattening phase are presented in Figure 3, stratified by the interaction among maternal feeding level, HT, and age. Glucose and fructosamine concentrations were generally similar between groups, except at 6 months of age, when values of T100%-HT and T100%-CONTROL bull calves were higher than those of T60%-HT bull calves (*p* < 0.05). Glucose concentrations of the bull calves during the fattening phase were positively correlated with fructosamine concentrations (r = 0.50, *p* < 0.001), reaching maximum correlation at 6 months of age (r = 0.85, *p* < 0.001). Likewise, glucose and fructosamine concentrations were positively correlated with LW of bull calves during the fattening phase (r = 0.41, *p* < 0.001, and r = 0.42, *p* < 0.001, respectively). Non-esterified fatty acid (NEFA) levels were similar between the groups. They decreased throughout fattening, except at 12 months of age, when the levels of the T60%-CONTROL and T60%-HT bull calves increased and tended to be higher than the levels of the T100%-CONTROL bull calves (*p* = 0.07 and *p* = 0.08, respectively). Urea levels fluctuated throughout the fattening phase in all groups. At 4 months of age, T60% bull calves (T60%-CONTROL and T60%-HT) had higher levels than T100%-HT bull calves (*p* < 0.05). However, from 6 months of age onwards, these differences disappeared until urea levels of T100%-CONTROL were higher than those of T100%-HT at month 10 (*p* < 0.05), and T60%-HT values were higher than those of T60%-CONTROL bull calves at month 12 (*p* < 0.05). Mean creatinine levels were lower in the T100%-CONTROL group in the second half of fattening, although no significant differences were observed between groups (*p* > 0.05). The urea/creatinine (U/C) ratio was higher in T60%-CONTROL than in T100%-CONTROL bull calves at 6 months of age (17.56 ± 1.58 vs. 12.05 ± 1.87, respectively; *p* < 0.05). This pattern was reversed at 10 months of age, when T100%-CONTROL bull calves showed a higher U/C ratio than T60%-CONTROL bull calves (28.65 ± 1.87 vs. 23.33 ± 1.58, respectively; *p* < 0.05). Regarding the plasma concentration of IGF-1, it increased from month 4 to 8 in all groups. Levels in the T100%-HT group tended to be lower than in the T60%-HT group at 6 months of age (*p* = 0.10). Insulin-like growth factor 1 levels during the fattening phase were positively correlated with glucose levels (r = 0.44, *p* < 0.001) and LW (r = 0.75, *p* < 0.001). Furthermore, the IGF-1 levels of the bull calves at different moments of the fattening phase were positively correlated with the IGF-1 levels of their dams during the last third of pregnancy (when maternal nutritional treatment was applied) [32]. Insulin-like growth factor 1 concentrations of bull calves at 4, 10 and 12 months of age were positively correlated with the IGF-1 concentrations of their dams at 40 weeks of gestation (after approximately 3 months of maternal nutritional treatment; r = 0.50, *p* < 0.001; r = 0.47, *p* < 0.001; and r = 0.33, *p* = 0.025, respectively).

## 4. Discussion

### 4.1. Concentrate Intake and Concentrate Conversion Ratio

Maternal feeding level showed a tendency to influence average concentrate intake and average conversion efficiency during the fattening phase. T60% bull calves tended to consume less concentrate and tended to have a lower concentrate conversion rate (i.e., greater efficiency) than T100% bull calves. Similarly, some studies suggest that maternal undernutrition during pregnancy may result in a lower feed conversion ratio in offspring, which is consistent with the “thrifty phenotype” theory [47,48]. Specifically, Nascimento et al. [47] observed that female calves from mothers with protein restriction had a higher feed conversion efficiency than those from supplemented mothers. Similarly, Nishino et al. [48] found that calves from nutritionally restricted mothers exhibited improved nutrient utilization efficiency during the post-weaning growth period, as evidenced by a trend toward lower conversion rates of crude protein (CP) and total digestible nutrients (TDN). This adaptation is interpreted as a programmed “energy-saving metabolism” in the offspring’s muscle, which suppresses energy production to conserve energy for muscle protein synthesis.

In this sense, other studies have observed that maternal undernutrition during pregnancy can lead to a higher feed conversion ratio in offspring, resulting in lower feed efficiency. For example, Noya et al. [29] reported that beef bull calves whose mothers were undernourished in the first third of pregnancy had a slightly higher average feed conversion rate compared to bull calves in the control group during the fattening phase. In this line, Nascimento et al. [47] found that, in males, calves from protein-restricted mothers at mid-gestation showed lower weight gain efficiency compared to males from supplemented mothers, which implied that males from undernourished mothers required more feed per unit of weight gain.

The contradictory findings regarding whether maternal undernutrition during pregnancy increases or decreases feed conversion ratio in bull calves can be explained by several biological and experimental factors. Differences in the timing of nutrient restriction during gestation (early vs. middle vs. late pregnancy), severity and duration of nutrient restriction, together with variations in the composition of fattening diets (crude protein, starch, fat content, and forage-to-concentrate ratio), postnatal management, season and climate during the fattening phase, genetic background, and other aspects of experimental design, are likely to underlie these discrepancies.

### 4.2. Bull Calf Growth and Development

Although average T60%-CONTROL bull calves’ weights were lower than those of T100%-CONTROL bull calves throughout fattening, these differences were not significant. In any case, maternal feeding level significantly affected body size measurements and BMI, as T60% bull calves were smaller and had lower BMI than T100% bull calves at the start of fattening, whereas no differences were observed at slaughter.

When evaluating fetal programming, it is important to acknowledge that postnatal maternal effects—such as colostrum quality and milk yield—can act as potential confounders for subsequent offspring growth. In a companion study evaluating the pre-weaning phase of this same herd (unpublished data from López de Armentia et al.), neither total milk yield nor calf milk intake were affected by the prepartum dietary treatments. Although maternal undernutrition influenced the overall weaning weight of the entire herd in that study, the specific sub-sample of male calves evaluated in the present fattening phase did not show significant differences in initial LW at four months of age, presenting only specific differences in some initial morphometric measurements.

Several studies have reported delayed growth in pre-weaning calves attributable to suboptimal maternal nutrition during the latter third of gestation. These studies have indicated that compensatory growth may be observed during the fattening phase and at slaughter, when high-quality diets and adequate food availability are ensured in the postnatal period [27,49,50]. This observation is consistent with the results of the present study. In contrast, other studies, such as Cafe et al. [51] and Greenwood et al. [52], have reported significant negative effects of maternal undernutrition during late pregnancy on offspring LW from birth to adulthood. The same effect was observed by Noya et al. [29] during the fattening phase of bull calves but, in this case, undernutrition was applied in the first third of gestation. The lower growth of calves from undernourished cows during fattening is due to the persistent effects of fetal programming, potentially involving impaired stem and satellite cell function [53]. In contrast, calves that experience compensatory growth may have benefited from favorable subsequent feeding conditions. However, results may differ due to variations in the timing, severity, and duration of undernutrition, as well as postnatal nutrition.

Furthermore, HT supplementation increased the LW of T60% bull calves during the second half of the fattening phase. Specifically, based on pairwise comparisons, T60%-CONTROL bull calves were about 31 kg lighter than their T60%-HT counterparts, corresponding to an approximate 6% reduction in final LW, whereas no effect of HT was observed in T100% bull calves. In a previous part of this study, as reported in López de Armentia et al. [32], a positive effect of maternal HT supplementation on calf birth weight was detected irrespective of the maternal feeding level (T100% vs. T60%), suggesting that HT improved prenatal growth in all calves but only translated into a sustained advantage during fattening under conditions of gestational undernutrition. It could be hypothesized that HT might exert its effects by potentially protecting against oxidative stress and enhancing metabolic efficiency in a fetal environment compromised by undernutrition. Although oxidative stress markers were not directly measured during the fattening phase in the present study, a companion study conducted on the same experimental herd [31] demonstrated that maternal HT supplementation successfully enhanced total plasma antioxidant capacity and upregulated antioxidant enzyme genes (such as SOD1, CAT, and GPX1) in the early postnatal life of calves born to undernourished dams. Therefore, it is plausible that this early-life redox protection programmed the offspring for improved long-term growth performance, even if these mechanisms remain speculative during the fattening phase. However, in well-fed cows, this effect may not be observed because the fetal environment is already optimal. Current literature lacks studies in cattle investigating the effect of HT during gestation on the development of male offspring during the fattening phase. Nevertheless, the impact of HT administration during gestation on offspring development has already been examined in pigs [24,54,55]. In the studies by Vazquez-Gomez et al. [24,38], maternal supplementation with HT had a positive effect on LW and ADG in pre-weaned and post-weaned piglets from undernourished dams. On the contrary, Gómez et al. [56] studied the effect of maternal supplementation with HT on the fattening phase of pigs, and observed no effect on the final LW of the offspring. It should be noted that the sows in that study were not undernourished, which is consistent with our findings that HT had no effect on offspring from non-undernourished mothers.

### 4.3. Metabolic and Endocrine Profiles

In general, maternal feeding level or HT did not have a significant impact on glucose profiles, indicating limited persistent differences in circulating glucose during the fattening phase. In this context, a previous study by this group found that maternal supplementation with HT improved glucose metabolism in T60% calves during the first week of life, as evidenced by enhanced transport and activation of gluconeogenic pathways [31]. However, this finding was insufficient to alter glucose levels in T60% bull calves during the fattening phase, as observed in the present study.

While blood glucose concentrations reflect the current state, serum fructosamine concentrations reflect the average glucose concentration over the preceding two to three weeks [57]. Fructosamine in cattle is an important biochemical parameter that has been studied primarily as an indicator of metabolic status, particularly for glycemic control and nutritional status [58,59]. In this regard, it has been observed that cows with chronic undernutrition or prolonged negative energy balance exhibit significantly lower serum fructosamine levels [58,60]. In the present study, the generally limited effects observed on fructosamine concentrations were consistent with the overall similarity in glucose profiles among experimental groups. Moreover, the parallel responses observed for both variables support the role of fructosamine as an integrated indicator of glucose status over time. Considering that blood samples were collected at two-month intervals, transient alterations in glucose metabolism may have been detected simultaneously in both glucose and fructosamine profiles, despite the different temporal window represented by each biomarker. Evidence regarding fructosamine profiles in beef bull calves born to dams subjected to maternal undernutrition is currently very limited. To our knowledge, no studies have specifically evaluated this biomarker in this context. Furthermore, the correlation between glucose and fructosamine levels has been studied in cattle, but results vary with the physiological and metabolic context. On the one hand, there is scientific evidence documenting the absence of a significant correlation between glucose and fructosamine, particularly in dynamic physiological situations such as the peripartum period [61]. On the other hand, research on calves indicates a positive correlation between fructosamine and glucose under stable metabolic conditions, as fructosamine serves as a cumulative marker of blood glucose levels over several weeks [62].

During the fattening phase, the decline in NEFA concentrations reflects a metabolic adaptation to increased energy intake, thereby limiting the mobilization of body fat as an energy source [63,64]. However, the tendency for higher NEFAs at 12 months in T60% bull calves could be hypothesized as a fetal programming effect leading to a thrifty phenotype. Nevertheless, since adiposity, insulin sensitivity, and lipid turnover were not directly measured in the present study, this interpretation remains speculative. If present, such a phenotype could theoretically maintain energy homeostasis by prioritizing adipose tissue mobilization even under ad libitum feeding, potentially due to altered sensitivity to hormones such as insulin [29,48]. At this stage, T60% and T100% bull calves in CONTROL groups showed similar concentrate intake and average daily gain. At the same time, the concentrate conversion ratio was numerically higher in T100% animals, although these differences were not statistically significant. In this context, the higher NEFA concentrations observed in T60% bull calves may be compatible with a compensatory growth response driven by programmed alterations in lipid metabolism, which could have implications for lipid and metabolic homeostasis in later phases of development [65].

Urea levels of T60% bull calves (T60%-CONTROL and T60%-HT) were similar between 6 and 10 months of age. However, at one year of age, T60%-HT bull calves exhibited elevated blood urea levels compared with T60%-CONTROL calves. This finding can be interpreted within the context of fetal programming. Previous studies have demonstrated that maternal undernutrition can induce a ‘thrifty’ nitrogen metabolism in the offspring, often characterized by the suppression of urea cycle-related genes to prioritize amino acids for tissue synthesis, leading to lower circulating urea [64,66]. Given that the estimated nitrogen use efficiency (the ratio of retained to consumed nitrogen) did not differ between these two restricted groups in the present study, the elevated plasma urea in T60%-HT calves is unlikely to reflect a higher rate of dietary protein degradation. Instead, it is hypothesized that maternal HT supplementation might have programmed a persistent physiological adaptation to optimize the urea salvaging pathway via the ruminal wall or saliva [67]. Since specific parameters such as total tract digestibility and nitrogen balance were not directly tested, this mechanism remains speculative, but it could potentially allow bull calves to maintain a robust nitrogen homeostasis within the gastrointestinal tract without altering their overall nitrogen utilization efficiency.

Creatinine levels were similar in all four groups throughout the fattening phase. However, when studying the U/C ratio, a biochemical indicator that reflects the relationship between nitrogen metabolism (urea) and muscle mass or renal function (creatinine), thus showing how efficiently the animal utilizes and eliminates metabolic nitrogen [68], significant differences were observed between the control groups (T100%-CONTROL and T60%-CONTROL). Although neither urea nor creatinine concentrations differed significantly between these two groups at the corresponding sampling times, their relationship, expressed as the U/C ratio, differed significantly. This finding may reflect temporal changes in the balance between urea and creatinine metabolism between T60% and T100% bull calves during the fattening phase, potentially associated with metabolic adaptations induced by prenatal nutritional restriction. Nevertheless, given the lack of significant differences in the individual metabolites and the reversal of the pattern over time, the biological relevance of these changes in the U/C ratio should be interpreted with caution.

Insulin-like growth factor type 1 (IGF-1) is a potent anabolic mediator that plays a key role in somatic growth, cell differentiation, and protein synthesis [69]. In the present study, no significant differences were observed in plasma IGF-1 concentrations between the four experimental groups. However, 60-CONTROL dams of the bull calves (used in the present experiment) showed the lowest IGF-1 levels during the last third of pregnancy [32], reflecting the impact of reduced energy intake on hepatic IGF-1 synthesis. Nonetheless, IGF-1 levels in bull calves correlated positively with glucose and LW, reinforcing its role as an indicator of the animals’ energy status and growth potential [70]. Although maternal supplementation with HT increased the LW and ADG of offspring from undernourished cows (T60%-HT bull calves), this effect was not accompanied by higher serum IGF-1 concentrations, suggesting that HT improved metabolic efficiency and glucose utilization via alternative pathways to the somatotropic axis. As previously described in cows from the same experimental herd, HT supplementation during late gestation increased the expression of genes involved in glucose metabolism in maternal blood cells. In addition, HT has been shown to attenuate oxidative stress and improve antioxidant capacity in the offspring, which may help preserve mitochondrial function and support more efficient glucose utilization in blood cells [31]. In line with these findings, the improved growth performance and the specific metabolic adaptations observed in T60%-HT bull calves (higher final LW and increased plasma urea at 12 months without major changes in glucose or IGF-1) generate the hypothesis that HT may primarily enhance metabolic efficiency and nutrient utilization through peripheral mechanisms (e.g., glucose and lipid metabolism, nitrogen recycling), rather than by a direct stimulation of IGF-1 secretion. However, as liver function, muscle histology, and mitochondrial or metabolic gene expression were not directly evaluated in the fattening bull calves, these mechanistic pathways remain hypothetical. Furthermore, positive correlations were observed between maternal IGF-1 concentrations during gestation (as reported in López de Armentia et al. [32]) and offspring IGF-1 concentrations throughout the fattening phase, indicating that dams with lower IGF-1 during pregnancy tended to produce calves with lower circulating IGF-1 and reduced growth performance. However, as previously mentioned, although differences in IGF-1 levels were observed between undernourished and non-undernourished cows [32], these differences were not reflected as consistent group differences in their offspring, suggesting that the observed correlations mainly reflect a shared underlying energy status rather than a persistent programming effect on the somatotropic axis itself.

## 5. Limitations

While this study provides valuable insights into the effects of maternal undernutrition and HT supplementation, several methodological constraints should be acknowledged. A primary limitation relates to the group-feeding structure. Because pregnant cows were housed in group pens, the exact individual ingestion of the compound from the TMR could not be assessed. However, to solve this constraint, cows were restrained at the feeder using self-locking headlocks for 3 h immediately after feed distribution to ensure equitable individual intake. Furthermore, the chemical stability of HT within the feed bunk was not continuously monitored, although adequate systemic absorption was confirmed in our companion study via blood and colostrum biomarkers [31,32].

Similarly, during the offspring’s fattening phase, concentrate intake and the resulting concentrate conversion ratio had to be recorded at the pen level (*n* = 4 pens per maternal feeding level), which limited the statistical power to assess individual feed efficiency. Furthermore, the sample size of 46 calves distributed across four treatment groups may have constrained the statistical precision to detect small treatment effects, with some variables showing statistical tendencies rather than reaching conventional levels of significance. Although environmental conditions were uniform across the barn, the inability to allocate calves into pens according to a full 2 × 2 factorial design due to space limitations means that potential pen-level social, feeding, or microenvironmental effects cannot be entirely ruled out and represent a methodological limitation. Furthermore, the reliance on unadjusted pairwise comparisons to explore specific treatment effects means that these particular findings should be interpreted with caution.

An additional limitation regarding feed efficiency is that voluntary barley straw intake was not directly quantified. Under our management conditions, straw was provided both as a forage source and as bedding material, making it unfeasible to accurately distinguish actual consumption from scattering due to normal exploratory behavior. Consequently, total dry matter intake and a complete feed conversion ratio could not be exactly calculated. To address this constraint, our analyses and results are exclusively based on the concentrate conversion ratio. While published data indicate that straw generally accounts for only 5–10% of total dry matter intake in concentrate-based beef fattening systems [40], potential variation among pens or over time cannot be excluded and should be considered when interpreting this parameter.

Additionally, the present follow-up during the fattening phase was conducted exclusively on male offspring. However, the evaluation of potential sex-specific programming effects on female progeny from this experimental cohort is currently being addressed in a separate companion study. Maternal HT supplementation was also evaluated during a single developmental window (the last third of gestation); future studies should explore whether extending supplementation throughout the entire pregnancy or first or second thirds might yield different postnatal outcomes.

Finally, it is important to acknowledge certain interpretative constraints regarding the physiological mechanisms discussed. Although nitrogen use efficiency was estimated based on concentrate intake and performance data, the lack of direct in vivo mechanistic measurements—such as specific hepatic metabolism, tissue-level insulin sensitivity, direct oxidative stress biomarkers during the fattening phase, mitochondrial function assays, or total tract digestibility—restricts our metabolic explanations to hypotheses rather than definitive conclusions.

## 6. Conclusions

Regarding offspring growth, maternal HT supplementation at 180 mg/kg diet did not show an overall effect on LW, although the specific comparison within the undernourished groups revealed that bull calves from HT-supplemented dams weighed 31 kg more at slaughter than their unsupplemented counterparts. This suggests that HT might be beneficial to amplify the compensatory growth of offspring facing prenatal nutritional challenges. In terms of body size, while maternal undernutrition reduced some bull calf morphometric measurements at the start of the fattening phase, these differences were no longer evident at slaughter, as optimal postnatal feeding allowed their recovery. Regarding physiological profiles, the higher plasma urea concentration observed in maternal HT-supplemented bull calves at 12 months of age suggests greater nitrogen turnover and adaptive metabolic efficiency under prior nutritional challenge. Taken together, maternal HT supplementation during late gestation represents a potential strategy to mitigate the adverse effects of prenatal undernutrition on offspring development, though further research into its long-term implications for sustainable beef cattle systems is needed.

## Figures and Tables

**Table 1:** Ingredients of the concentrate (g/100 g, as-fed basis).

Ingredient	g/100 g
Corn	44
Barley 8% CP	21.6
Gluten feed 19% CP	15
Rapeseed meal 34% CP	5
Soybean meal 47% CP	4.6
Sugarbeet pulp	3
Palm oil	2.9
Calcium carbonate	1.2
Urea	0.5
Sodium chloride	0.2
Vitamin–mineral premix *	2

* Vitamin–mineral complex (composition per kg of feed): 6000 IU of Vitamin A, 1200 IU of Vitamin D3, 10 mg of Vitamin E, 20 mg of Fe (ferrous carbonate), 2 mg of I (calcium iodate), 0.2 mg of Co (cobaltous carbonate), 30 mg of Mn (manganese oxide), 50 mg of Zn (zinc oxide), 2 mg of Se (sodium selenite), 0.03 mg of citric acid, 0.12 mg of butyl-hydroxytoluene (BHT) and 0.5 g of sepiolite. CP = crude protein.

**Table 2:** Chemical composition and energy values of feedstuffs (g/kg DM, dry matter basis).

Chemical Composition	Concentrate	Barley Straw *
DM (g/kg)	881	914
CP (g/kg DM)	141	46
EE (g/kg DM)	58	9
NDF (g/kg DM)	204	711
ADF (g/kg DM)	70	457
ADL (g/kg DM)	11	84
Ash (g/kg DM)	60	69
NE for meat production (MJ/kg DM) ^†^	8.0	2.3

* Chemical composition based on FEDNA tables [41]. ^†^ Net energy for meat production was estimated as the weighted sum of the net energy value of each ingredient according to the FEDNA tables [41]. DM = dry matter; CP = crude protein; EE = ether extract; NDF = neutral-detergent fiber; ADF = acid-detergent fiber; ADL = acid-detergent lignin; NE = net energy.

**Table 3:** Growth performance of bull calves during the fattening phase according to maternal feeding level (FL) and hydroxytyrosol supplementation (HT).

	FL × HT					*p*-Value		
	T100%- CONTROL	T60%- CONTROL	T100%- HT	T60%- HT	RSD	FL	HT	FL × HT
*n*	10	13	11	12				
Fattening phase (days)	235	236	237	235	2.84	0.6794	0.619	0.120
Age at slaughter (days)	353	353	356	356	6.20	0.916	0.137	0.977
Initial LW (kg) *	164	144	162	162	15.51	0.323	0.252	0.084
Final LW (kg) *	503	484	493	515	15.51	0.323	0.252	0.084
ADG (kg/d)	1.54	1.55	1.57	1.65	0.16	0.534	0.670	0.619

* For LW, *p*-values for FL, HT, and FL × HT were obtained from the repeated-measures mixed model including all sampling times; therefore, they represent overall effects across the fattening phase and are not specific to initial or final LW. Although the overall FL × HT interaction showed a tendency (*p* = 0.084), pairwise comparisons at 12 months revealed that T60%-HT bull calves had a higher final LW than T60%-CONTROL bull calves (515 vs. 484 kg; *p* = 0.023). T100%-CONTROL = bull calves from cows fed 100% of their requirements and not supplemented with HT; T60%-CONTROL = bull calves from cows fed 60% of their requirements and not supplemented with HT; T100%-HT = bull calves from cows fed 100% of their requirements and supplemented with HT; T60%-HT = bull calves from cows fed 60% of their requirements and supplemented with HT; RSD = residual standard deviation; LW = live weight; ADG = average daily gain.

**Table 4:** Body size measures of bull calves at the start (4 months of age) and at the end of the fattening phase (12 months of age), according to maternal feeding level (FL), hydroxytyrosol supplementation (HT), and breed.

	FL		HT		Breed			*p*-Value		
	T100%	T60%	CONTROL	HT	PA	PI	RSD	FL	HT	Breed
*n*	21	25	23	23	25	21				
Height at withers (cm)										
4 months	96.9	95.9	95.9	96.9	96.2	96.6	2.73	0.123	0.860	0.574
12 months	121	122	121	122	122	122	2.90	0.836	0.774	0.991
Height at rump (cm)										
4 months	103 ^a^	101 ^b^	102	102	102	103	2.76	0.015	0.367	0.259
12 months	129	128	128	129	128	129	3.44	0.628	0.734	0.271
Rump width (cm)										
4 months	23.2	23.1	23.1	23.2	23	23.3	1.75	0.904	0.806	0.641
12 months	44.5	44.6	44.3	44.8	45.6 ^a^	43.5 ^b^	3.03	0.431	0.673	0.020
Rump length (cm)										
4 months	32.7	32.6	32.8	32.5	32.3	33	1.66	0.982	0.379	0.212
12 months	47.8	46.6	47.2	47.1	47.2	47.2	2.69	0.159	0.352	0.973
Body length (cm)										
4 months	100.6 ^a^	97.9 ^b^	99.2	99.2	99.2	99.3	4.14	0.027	0.921	0.915
12 months	136	135	134	137	136	135	6.52	0.464	0.628	0.543
Chest girth (cm)										
4 months	122 ^a^	119 ^b^	120	120	122	119	4.79	0.004	0.495	0.083
12 months	188	187	187	188	190 ^a^	185 ^b^	6.06	0.137	0.651	0.005
Head girth (cm)										
4 months	73.1 ^a^	71.3 ^b^	72.4	72	72.7	71.6	3.13	0.019	0.290	0.205
12 months	106	109	107	109	109	107	5.58	0.317	0.690	0.278
Metacarpal girth (cm)										
4 months *	15.4	15.5	15.7 ^a^	15.2 ^b^	15.8 ^a^	15.2 ^b^	0.75	0.707	0.022	0.007
12 months	22.1	22.5	22.3	22.3	22.7 ^a^	21.8 ^b^	0.98	0.638	0.426	0.003

* The interaction between FL and HT only affected metacarpal girth at the initial stage of the fattening phase (16.0 ^a^, 15.4 ^ab^, 14.9 ^b^ and 15.6 ^a^ for T100%-CONTROL, T60%-CONTROL, T100%-HT and T60%-HT, respectively; *p* = 0.007). T100% = bull calves from cows fed 100% of their requirements during the last third of pregnancy; T60% = bull calves from cows fed 60% of their requirements during the last third of pregnancy; CONTROL = bull calves from cows not supplemented with HT; HT = bull calves from cows supplemented with HT; PA = Parda de Montaña; PI = Pirenaica; RSD = residual standard deviation. ^a,b^ Means within a row differ, *p* < 0.05.

## Data Availability

The data supporting the study findings will be available in public, FAIR-compliant repositories (CITAREA and ZENODO). Until then, data are available from the authors upon reasonable request.

## References

[1] Greenwood P.L., Cafe L.M. (2007). Prenatal and Pre-Weaning Growth and Nutrition of Cattle: Long-Term Consequences for Beef Production. Animal.

[2] Oksbjerg N., Gondret F., Vestergaard M. (2004). Basic Principles of Muscle Development and Growth in Meat-Producing Mammals as Affected by the Insulin-like Growth Factor (IGF) System. Domest. Anim. Endocrinol..

[3] Maltin C.A., Delday M.I., Sinclair K.D., Steven J., Sneddon A.A. (2001). Impact of Manipulations of Myogenesis in Utero on the Performance of Adult Skeletal Muscle. Reproduction.

[4] Du M., Tong J., Zhao J., Underwood K.R., Zhu M., Ford S.P., Nathanielsz P.W. (2010). Fetal Programming of Skeletal Muscle Development in Ruminant Animals. J. Anim. Sci..

[5] Funston R.N., Larson D.M., Vonnahme K.A. (2010). Effects of Maternal Nutrition on Conceptus Growth and Offspring Performance: Implications for Beef Cattle Production. J. Anim. Sci..

[6] Reynolds L.P., Borowicz P.P., Caton J.S., Vonnahme K.A., Luther J.S., Hammer C.J., Maddock Carlin K.R., Grazul-Bilska A.T., Redmer D.A. (2010). Developmental Programming: The Concept, Large Animal Models, and the Key Role of Uteroplacental Vascular Development. J. Anim. Sci..

[7] Kamath U., Rao G., Kamath S.U., Rai L. (2006). Maternal and Fetal Indicators of Oxidative Stress during Intrauterine Growth Retardation (IUGR). Indian J. Clin. Biochem..

[8] Biri A., Bozkurt N., Turp A., Kavutcu M., Himmetoglu Ö., Durak I. (2007). Role of Oxidative Stress in Intrauterine Growth Restriction. Gynecol. Obstet. Investig..

[9] Gupta P., Narang M., Banerjee B.D., Basu S. (2004). Oxidative Stress in Term Small for Gestational Age Neonates Born to Undernourished Mothers: A Case Control Study. BMC Pediatr..

[10] Parraguez V.H., Atlagich M., Araneda O., García C., Muñoz A., De Los Reyes M., Urquieta B. (2011). Effects of Antioxidant Vitamins on Newborn and Placental Traits in Gestations at High Altitude: Comparative Study in High and Low Altitude Native Sheep. Reprod. Fertil. Dev..

[11] Parraguez V.H., Urquieta B., De Los Reyes M., González-Bulnes A., Astiz S., Muñoz A. (2013). Steroidogenesis in Sheep Pregnancy with Intrauterine Growth Retardation by High-Altitude Hypoxia: Effects of Maternal Altitudinal Status and Antioxidant Treatment. Reprod. Fertil. Dev..

[12] Sales F., Peralta O.A., Narbona E., McCoard S., Lira R., De Los Reyes M., González-Bulnes A., Parraguez V.H. (2019). Maternal Supplementation with Antioxidant Vitamins in Sheep Results in Increased Transfer to the Fetus and Improvement of Fetal Antioxidant Status and Development. Antioxidants.

[13] Parraguez V.H., Sales F., Peralta O.A., Narbona E., Lira R., De Los Reyes M., González-Bulnes A. (2020). Supplementation of Underfed Twin-Bearing Ewes with Herbal Vitamins C and E: Impacts on Birth Weight, Postnatal Growth, and Pre-Weaning Survival of the Lambs. Animals.

[14] Gessner D.K., Ringseis R., Eder K. (2017). Potential of Plant Polyphenols to Combat Oxidative Stress and Inflammatory Processes in Farm Animals. J. Anim. Physiol. Anim. Nutr..

[15] Ly C., Yockell-Lelièvre J., Ferraro Z.M., Arnason J.T., Ferrier J., Gruslin A. (2015). The Effects of Dietary Polyphenols on Reproductive Health and Early Development. Hum. Reprod. Update.

[16] Prior R.L., Gu L., Wu X., Jacob R.A., Sotoudeh G., Kader A.A., Cook R.A. (2007). Plasma Antioxidant Capacity Changes Following a Meal as a Measure of the Ability of a Food to Alter in Vivo Antioxidant Status. J. Am. Coll. Nutr..

[17] Chen B., Tuuli M.G., Longtine M.S., Shin J.S., Lawrence R., Inder T., Nelson D.M. (2012). Pomegranate Juice and Punicalagin Attenuate Oxidative Stress and Apoptosis in Human Placenta and in Human Placental Trophoblasts. Am. J. Physiol. Endocrinol. Metab..

[18] Leslie M. (2010). Reactive Oxygen and Nitrogen Species and Functional Adaptation of the Placenta. Placenta.

[19] Jauniaux E., Burton G.J. (2016). Le Rôle Du Stress Oxydant Dans Les Pathologies Placentaires de La Grossesse. J. Gynecol. Obstet. Biol. Reprod..

[20] Burton G.J., Yung H.W., Cindrova-Davies T., Charnock-Jones D.S. (2009). Placental Endoplasmic Reticulum Stress and Oxidative Stress in the Pathophysiology of Unexplained Intrauterine Growth Restriction and Early Onset Preeclampsia. Placenta.

[21] Rigacci S., Stefani M. (2016). Nutraceutical Properties of Olive Oil Polyphenols. An Itinerary from Cultured Cells through Animal Models to Humans. Int. J. Mol. Sci..

[22] Fang X., Cao J., Tao Z., Yang Z., Dai Y., Zhao L. (2023). Hydroxytyrosol Attenuates Ethanol-Induced Liver Injury by Ameliorating Steatosis, Oxidative Stress and Hepatic Inflammation by Interfering STAT3/INOS Pathway. Redox Rep..

[23] Tundis R., Loizzo M., Menichini F., Statti G., Menichini F. (2008). Biological and Pharmacological Activities of Iridoids: Recent Developments. Mini-Rev. Med. Chem..

[24] Vázquez-Gómez M., Heras-Molina A., García-Contreras C., Pesantez-Pacheco J.L., Torres-Rovira L., Martínez-Fernández B., González J., Encinas T., Astiz S., Ovilo C. (2019). Polyphenols and IUGR Pregnancies: Effects of Maternal Hydroxytyrosol Supplementation on Postnatal Growth, Metabolism and Body Composition of the Offspring. Antioxidants.

[25] García-Contreras C., Vázquez-Gómez M., Barbero A., Pesantez J.L., Zinellu A., Berlinguer F., González-Añover P., González J., Encinas T., Torres-Rovira L. (2019). Polyphenols and IUGR Pregnancies: Effects of Maternal Hydroxytyrosol Supplementation on Placental Gene Expression and Fetal Antioxidant Status, DNA-Methylation and Phenotype. Int. J. Mol. Sci..

[26] Kang K., Zeng L., Ma J., Shi L., Hu R., Zou H., Peng Q., Wang L., Xue B., Wang Z. (2022). High Energy Diet of Beef Cows during Gestation Promoted Growth Performance of Calves by Improving Placental Nutrients Transport. Front. Vet. Sci..

[27] Klein J.L., Adams S.M., Moura A.F., Borchate D., Alves Filho D.C., Antunes D.P., Maidana F.M., Cardoso G.S., Brondani I.L., Gindri R.G. (2022). Effect of Nutrition in the Final Third of Gestation of Beef Cows on Progeny Development. Rev. Mex. Cienc. Pecu..

[28] Noya A., Casasús I., Ferrer J., Sanz A. (2019). Effects of Developmental Programming Caused by Maternal Nutrient Intake on Postnatal Performance of Beef Heifers and Their Calves. Animals.

[29] Noya A., Ripoll G., Casasús I., Sanz A. (2022). Long-Term Effects of Early Maternal Undernutrition on the Growth, Physiological Profiles, Carcass and Meat Quality of Male Beef Offspring. Res. Vet. Sci..

[30] Polizel G.H.G., dos Santos M.E.P., Cesar A.S.M., Diniz W.J.S., Ramírez-Zamudio G.D., Fantinato-Neto P., Fernandes A.C., Prati B.C.T., Furlan É., Pombo G.d.V. (2025). Late Gestation Maternal Nutrition Has a Stronger Impact on Offspring Liver Transcriptome than Full-Gestation Supplementation in Beef Cattle. Vet. Sci..

[31] Escalera-Moreno N., Álvarez-Rodríguez J., López de Armentia L., Macià A., Martín-Alonso M.J., Molina E., Villalba D., Sanz A., Serrano-Pérez B. (2025). Maternal Hydroxytyrosol Supplementation Enhances Antioxidant Capacity and Immunometabolic Adaptations in Nutrient-Restricted Beef Cows and Their Offspring. Antioxidants.

[32] López de Armentia L., Noya A., Álvarez-Rodríguez J., Villalba D., Serrano-Pérez B., Casasús I., Alabart J.L., Sanz A. (2026). Effects of Undernutrition and Hydroxytyrosol Supplementation in Late Pregnancy on Cow-Calf Performance, Metabolic and Immune Status, and Newborn Vitality in Beef Herds. Animal.

[33] Escalera-Moreno N., Serrano-Pérez B., Blanco-Penedo I., López de Armentia L., Noya A., Sanz A., Álvarez-Rodríguez J. (2026). Effects of Late-Gestation Nutritional Restriction and Hydroxytyrosol Supplementation on Behavioural Responses and Neuroendocrine Blood Markers in Beef Cows and Their Calves. Agriculture.

[34] Ministerio de Agricultura Pesca y Alimentación (MAPA) (2025). Informe Resumen de Caracterización Del Sector Vacuno de Carne. Datos Año 2024.

[35] INRA, INRA (2018). INRA Feeding System for Ruminants.

[36] Robles-Almazan M., Pulido-Moran M., Moreno-Fernández J., Ramirez-Tortosa C., Rodriguez-Garcia C., Quiles J.L. (2018). Hydroxytyrosol: Bioavailability, Toxicity, and Clinical Applications. Food Res. Int..

[37] Rodríguez-Gutiérrez G., Duthie G.G., Wood S., Morrice P., Nicol F., Reid M., Cantlay L.L., Kelder T., Horgan G.W., Fernández-Bolaños J. (2012). Alperujo Extract, Hydroxytyrosol, and 3,4-Dihydroxyphenylglycol Are Bioavailable and Have Antioxidant Properties in Vitamin E-Deficient Rats—A Proteomics and Network Analysis Approach. Mol. Nutr. Food Res..

[38] Vázquez-Gómez M., García-Contreras C., Torres-Rovira L., Pesantez J.L., González-Añover P., Gómez-Fidalgo E., Sánchez-Sánchez R., Ovilo C., Isabel B., Astiz S. (2017). Polyphenols and IUGR Pregnancies: Maternal Hydroxytyrosol Supplementation Improves Prenatal and Early-Postnatal Growth and Metabolism of the Offspring. PLoS ONE.

[39] Turck D., Bresson J., Burlingame B., Dean T., Fairweather-Tait S., Heinonen M., Hirsch-Ernst K.I., Mangelsdorf I., McArdle H.J., Naska A. (2017). Scientific Opinion on the Safety of Hydroxytyrosol as a Novel Food Pursuant to Regulation (EC) No 258/97. EFSA J..

[40] Arbaoui A., de Vega A. (2023). Does Replacing Maize with Barley Affect the Animal Performance and Rumen Fermentation, Including Methane Production, of Beef Cattle Fed High-Concentrate Diets On-Farm?. Animals.

[41] De Blas C., García-Rebollar P., Gorrachategui M., Mateos G.G. (2019). Tablas FEDNA de Composición y Valor Nutritivo de Alimentos para la Fabricación de Piensos Compuestos.

[42] Ministerio de Agricultura Pesca y Alimentación (MAPA) (2019). Bases Zootécnicas para el Cálculo del Balance Alimentario de Nitrogeno y de Fósforo.

[43] Maresca S., López Valiente S., Rodríguez A.M., Long N.M., Pavan E., Quintans G. (2018). Effect of Protein Restriction of Bovine Dams during Late Gestation on Offspring Postnatal Growth, Glucose-Insulin Metabolism and IGF-1 Concentration. Livest. Sci..

[44] R Core Team (2024). R: A Language and Environment for Statistical Computing.

[45] Kuznetsova A., Brockhoff P.B., Christensen R.H.B. (2017). LmerTest Package: Tests in Linear Mixed Effects Models. J. Stat. Softw..

[46] Lenth R., Piaskowski J. Emmeans: Estimated Marginal Means, Aka Least-Squares Means. R Package Version 2.0.0. https://rvlenth.github.io/emmeans/.

[47] Nascimento K.B., Galvão M.C., Meneses J.A.M., Moreira G.M., Ramírez-Zamudio G.D., de Souza S.P., Prezotto L.D., Chalfun L.H.L., Duarte M.D.S., Casagrande D.R. (2022). Effects of Maternal Protein Supplementation at Mid-Gestation of Cows on Intake, Digestibility, and Feeding Behavior of the Offspring. Animals.

[48] Nishino D., Haginouchi T., Shimogiri T., Muroya S., Kawabata K., Urasoko S., Oshima I., Yasuo S., Gotoh T. (2025). A Pilot Study: Maternal Undernutrition Programs Energy Metabolism and Alters Metabolic Profile and Morphological Characteristics of Skeletal Muscle in Postnatal Beef Cattle. Metabolites.

[49] Klein J.L., Adams S.M., Alves Filho D.C., Brondani I.L., Pizutti L.Â.D., Cocco J.M. (2023). Effects of Maternal Nutrition in the Final Third of Gestation on Performance and Body Composition of Progeny at Slaughter. Ciên. Anim. Bras..

[50] Ramírez M., Testa L.M., López Valiente S., Latorre M.E., Long N.M., Rodríguez A.M., Pavan E., Maresca S. (2020). Maternal Energy Status during Late Gestation: Effects on Growth Performance, Carcass Characteristics and Meat Quality of Steers Progeny. Meat Sci..

[51] Cafe L.M., Hennessy D.W., Hearnshaw H., Morris S.G., Greenwood P.L. (2009). Consequences of Prenatal and Preweaning Growth for Feedlot Growth, Intake and Efficiency of Piedmontese- and Wagyu-Sired Cattle. Anim. Prod. Sci..

[52] Greenwood P.L., Cafe L.M., Hearnshaw H., Hennessy D.W., Thompson J.M., Morris S.G. (2006). Long-Term Consequences of Birth Weight and Growth to Weaning on Carcass, Yield and Beef Quality Characteristics of Piedmontese- and Wagyu-Sired Cattle. Aust. J. Exp. Agric..

[53] Hoffman M.L., Reed S.A., Pillai S.M., Jones A.K., McFadden K.K., Zinn S.A., Govoni K.E. (2017). PHYSIOLOGY AND ENDOCRINOLOGY SYMPOSIUM: The Effects of Poor Maternal Nutrition during Gestation on Offspring Postnatal Growth and Metabolism. J. Anim. Sci..

[54] Laviano H.D., Gómez G., Núñez Y., García-Casco J.M., Benítez R.M., de las Heras-Molina A., Gómez F., Sánchez-Esquiliche F., Martínez-Fernández B., González-Bulnes A. (2024). Maternal Dietary Antioxidant Supplementation Regulates Weaned Piglets’ Adipose Tissue Transcriptome and Morphology. PLoS ONE.

[55] García-Contreras C., Vázquez-Gómez M., Pardo Z., Heras-Molina A., Pesantez J.L., Encinas T., Torres-Rovira L., Astiz S., Nieto R., Ovilo C. (2019). Polyphenols and IUGR Pregnancies: Effects of Maternal Hydroxytyrosol Supplementation on Hepatic Fat Accretion and Energy and Fatty Acids Profile of Fetal Tissues. Nutrients.

[56] Gómez G., Laviano H.D., García-Casco J., Muñoz M., Gómez F., Sánchez-Esquiliche F., González-Bulnes A., López-Bote C., Óvilo C., Rey A.I. (2024). Long-Term Effect of Maternal Antioxidant Supplementation on the Lipid Profile of the Progeny According to the Sow’s Parity Number. Antioxidants.

[57] Armbruster D.A. (1987). Fructosamine: Structure, Analysis, and Clinical Usefulness. Clin. Chem..

[58] Jensen A.L., Petersen M.B., Houe H. (1993). Determination of the Fructosamine Concentration in Bovine Serum Samples. J. Vet. Med. Ser. A.

[59] Ropstad E. (1987). Serum Fructosamine Levels in Dairy Cows Related to Metabolic Status in Early Lactation. Acta Vet. Scand..

[60] Strydom S., Agenas S., Heath M.F., Phillips C.J.C., Rautenbach G.H., Thompson P.N. (2008). Evaluation of Biochemical and Ultrasonographic Measurements as Indicators of Undernutrition in Cattle. Onderstepoort J. Vet. Res..

[61] Ceballos A., Gómez P.M., Vélez M.L., Villa N.A., López L.F. (2002). Variación de Los Indicadores Bioquímicos Del Balance de Energía Según El Estado Productivo En Bovinos Lecheros de Manizales, Colombia. Rev. Colomb. Cienc. Pecu..

[62] Coppo J.A. (2001). Evolution of Fructosaminaemia and Glucaemia during the Growth of Unweaned and Early Weaned Half-Bred Zebu Calves. Vet. Res. Commun..

[63] Fox M.T., Gerrelli D., Pitt S.R., Jacobs D.E. (1991). The Relationship between Appetite and Plasma Non-Esterified Fatty Acid Levels in Housed Calves. Vet. Res. Commun..

[64] Redifer C.A., Wichman L.G., Davies-Jenkins S.L., Rathert-Williams A.R., Freetly H.C., Meyer A.M. (2024). Late Gestational Nutrient Restriction in Primiparous Beef Females: Performance and Metabolic Status of Lactating Dams and Pre-Weaning Calves. J. Anim. Sci..

[65] Long J.M., Trubenbach L.A., Pryor J.H., Long C.R., Wickersham T.A., Sawyer J.E., Satterfield M.C. (2021). Maternal Nutrient Restriction Alters Endocrine Pancreas Development in Fetal Heifers. Domest. Anim. Endocrinol..

[66] Muroya S., Zhang Y., Otomaru K., Oshima K., Oshima I., Sano M., Roh S., Ojima K., Gotoh T. (2022). Maternal Nutrient Restriction Disrupts Gene Expression and Metabolites Associated with Urea Cycle, Steroid Synthesis, Glucose Homeostasis, and Glucuronidation in Fetal Calf Liver. Metabolites.

[67] Batista E.D., Detmann E., Titgemeyer E.C., Valadares Filho S.C., Valadares R.F.D., Prates L.L., Rennó L.N., Paulino M.F. (2016). Effects of Varying Ruminally Undegradable Protein Supplementation on Forage Digestion, Nitrogen Metabolism, and Urea Kinetics in Nellore Cattle Fed Low-Quality Tropical Forage. J. Anim. Sci..

[68] Paulus M.C., Melchers M., van Es A., Kouw I.W.K., van Zanten A.R.H. (2025). The Urea-to-Creatinine Ratio as an Emerging Biomarker in Critical Care: A Scoping Review and Meta-Analysis. Crit. Care.

[69] Werner H. (2023). The IGF1 Signaling Pathway: From Basic Concepts to Therapeutic Opportunities. Int. J. Mol. Sci..

[70] Bai F., Cai Y., Qi M., Liang C., Pan L., Liu Y., Feng Y., Cao X., Yang Q., Ren G. (2025). LCORL and STC2 Variants Increase Body Size and Growth Rate in Cattle and Other Animals. Genom. Proteom. Bioinform..

